# Looking to the (far) future of climate projection

**DOI:** 10.1111/gcb.15936

**Published:** 2021-10-24

**Authors:** Chris A. Boulton

**Affiliations:** ^1^ Global Systems Institute University of Exeter Exeter UK

The use of climate projections is important in determining Earth's future and our place in it. These projections inform policy, both at the country scale and globally, assisting leaders in their commitment to various pledges of mitigation in an attempt to prevent further climate change in the future and maybe even reverse it. The Intergovernmental Panel on Climate Change (IPCC) releases periodic ‘Assessment Reports’ which provide a current state of the climate and climate projections from modelling centres. The IPCC’s 5th Assessment Report, published in 2015, helped leaders shape the Paris Agreement. We are currently in the midst of the publication of their 6th report, AR6, the first volume of which will strongly influence the 2021 United Nations Climate Change Conference (COP26), with the other two volumes being published afterwards.

Climate projections are formed from the output of general circulation models (GCMs) created by climate research centres, and consist of modules that model certain aspects of the climate (e.g. the atmosphere, oceans, vegetation). These are then combined to model Earth's overall climate. Using models from a variety of centres allows uncertainty in the Earth processes to be explored due to the differences in the equations and parameters driving each GCM. Consequently, projections provide outputs from which a range of plausible outcomes can be determined, such as global mean temperature or changes in precipitation. Within each GCM, further uncertainty can also be explored by altering the specific variables in the equations that describe each aspect of the climate. These ‘perturbed‐parameter ensemble’ experiments provide valuable information about the spread of possible outcomes for a given GCM.

Furthermore, scenarios of emissions are also supplied to GCMs to explore the consequences of differing levels of human influence. Included in these are storylines illustrating how climate change can be mitigated, or adapted to, with inputs supplied to the GCMs, such as the amount of fossil fuel burning or the uptake of green energy, adjusted accordingly to alter the projection. The models contributing to AR6 are driven by scenarios known as Shared Socioeconomic Pathways (SSPs; Riahi et al., 2017). There are five main SSPs, each focusing on a separate narrative described by the socio‐economic challenges to either adaptation or mitigation of climate change. SSP1, for example, focuses on sustainability, with low challenges to both mitigation and adaptation (such that either are easily achieved). In this case, there is a shift from focusing on economic growth, to an emphasis on human wellbeing. SSP5, on the other hand, paints a picture of ‘Fossil‐fueled Development’, where challenges to mitigation are increased and a focus is placed on capital, investment and technology. In AR6, sub‐categories of the scenarios have been used and ranked according to the amount of radiative forcing (the change in energy flux in the atmosphere) they create by 2100. In IPCC terminology, at the lower end of the scale under SSP1‐1.9 (where global CO_2_ emissions become net zero by 2050), models predict a ‘very likely’ (90% or greater probability, conditional on this specific scenario) range of 0.15–0.95°C of global temperature increase by 2081–2100, relative to 1995–2014. In comparison, models driven by SSP5‐8.5 (where global CO_2_ emissions roughly double compared to current levels) predict a ‘very likely’ increase of 2.45–4.85°C by the end of the century (IPCC, 2021).

When the 1st Assessment Report (FAR) was created in 1990, the scenarios within ran to 2100. Since then, this has generally become an arbitrary fixed threshold that climate projections are run to. However, increases in anthropogenic emissions since then, along with advances in understanding climate change, have made it clear that changes past 2100 are just as influential in Earth's future, something that Lyon et al. (2021) highlight in this issue. Climate tipping points, where changes in the external forcing of a system could cause large, often irreversible changes to its stability are possible (Lenton et al., 2008), without any changes in the system observed by 2100. One such tipping point concerns the dieback of the Amazon rainforest, but only in a few models is this observed by the end of the century due to drier conditions; in other models, the Amazon appears to remain stable, or even grow in biomass (Huntingford et al., 2013). However, due to the slow dynamics of the vegetation, the forest may already be ‘committed’ to dieback, even if it appears stable in 2100 (Jones et al., 2009). Indeed, model studies have shown that without further emissions past 2100, allowing the climate system and in turn the Amazon rainforest to reach an equilibrium state, the condition of the forest could be greatly degraded (Boulton et al., 2017). With the rainforest acting as the world's largest terrestrial sink, forest loss will have a huge impact on the global carbon cycle and thus future climate change.

Another example of a potential climate tipping point, the collapse of the Atlantic Meridional Overturning Circulation (AMOC), is deemed ‘very unlikely’ (10% or less probability) to occur by 2100, although it is still likely that the AMOC will weaken over this time (IPCC, 2021). A single model experiment suggests a full AMOC collapse would cause large temperature decreases across the northern hemisphere, 1–3°C in North America and 3–8°C across Europe, and a southward shift of the Intertropical Convergence Zone (ITCZ) would cause drying across most of the northern hemisphere (Jackson et al., 2015). AMOC collapse is considered by the IPCC using GCMs run up to 2300, but these results are separate from the more in‐depth 21st century projections. In these runs, collapse has become ‘as likely as not’ for higher emissions pathways by 2300 (IPCC, 2019), and as such, consequences like these should be considered in decision‐making, particularly if they become even more likely in the further future.

Considering the potential for this longer‐term climate change, Lyon et al. (2021) look past the 2100 horizon while focusing on specific issues that only begin to appear in projections by the end of the century. They have run a model up to 2500 which provides a range of responses using the Representative Concentration Pathway (RCP) scenarios, precursors to the SSPs, and that were used in the 5th Assessment Report projections. RCP scenarios have storylines that run to 2300 (after which radiative forcing remains constant), but these are still yet to be fully utilized due to the computational power needed to run them. It is clear from Lyon et al.’s results though that global mean temperature increases into future centuries should also be part of the considerations for present‐day decision‐makers. These temperatures, even under reasonable mitigation, can have highly aversive effects on human health and the ability to grow crops, at least those currently grown. The effects suggest that adaptation to climate change will play a strong role in far‐future life.

Adding to the projections from their model runs are striking artist impressions of what certain aspects of the future living will look like, showing how the Amazon rainforest might look in the future, potentially needed adaptations to crop growing practices, and how people may survive in extreme temperatures in certain regions of the world.

Of course, some caution should be considered when looking at a single GCM run to make future projections, especially when considering the range of responses from a perturbed‐parameter ensemble of the same model found in a previous experiment by 2100 (Booth et al., 2013). Climate projections made from a suite of models provide an element of uncertainty that is otherwise missing when only considering uncertainties from scenarios. Nonetheless, Lyon et al.’s work highlights the importance of looking further than the end of the century when assessing what can be actioned now. It is clear that climate change and its related impacts generally become more severe after the 21st century, even if the specific details would differ between GCMs. With computing power ever increasing, alongside the current state of the climate, it is now time to look beyond the 2100 horizon.
